# A Study of Chemical Substances Migrated from Plastic Tableware to Evaluate the Food Safety for Pets

**DOI:** 10.5702/massspectrometry.A0119

**Published:** 2023-04-06

**Authors:** Yamato Miyazaki, Atsushi Yamamoto

**Affiliations:** 1 Faculty of Environment, Tottori University of Environmental Studies, 1–1–1 Wakabadaikita, Tottori, Tottori 689–1111, Japan

**Keywords:** food safety, pet, liquid chromatography/mass spectrometry, photoionization

## Abstract

To evaluate the safety of food for pets, the migration of chemical substances from pet tableware was investigated by mass spectrometry. The presence of polymer additives Irgafos 168 and Erucamide were suspected based on mass spectra and were confirmed to be present in polypropylene tableware. The amount of substances migrated using simulated saliva was examined by liquid chromatography-mass spectrometry after solid phase extraction and purification. Photoionization was found to be suitable for the simultaneous determination of these substances. The detection limits of the established method were 0.019 μg/mL for Irgafos 168 and 0.022 μg/mL for Erucamide. Five different types of pet tableware purchased in local markets were examined and no analytes were detected in the simulated saliva using shaking extraction. In this study, the risk to pets from the substances migrated from pet tableware was considered to be sufficiently low.

## INTRODUCTION

Plastics are used in a wide range of fields, ranging from industrial applications to commodities, and are one of the essential materials in use in today’s world. Additives are added to most plastic products to preserve the properties of the polymer substrates and to provide new functionalities. Among plastics, interest in plastics used with food products is growing worldwide because they can come into oral contact. From the viewpoint of safety and environmental protection, the use of additives that have adverse effects has been restricted. In the USA, EU, and Japan, lists of acceptable materials for use in food contact materials have been introduced. Positive list regulation is a framework that prohibits the use of substances other than those that have been approved. In Japan, the positive list regulation for food utensils, containers, and packaging came into effect in June 2020. The target substances include polymer substrates and additives. Such substances are classified into five categories under consideration as of 2022, and the amount to be used is determined according to the categories. Many studies have appeared regarding the safety of plastic food utensils, containers, and packaging. Additives that migrate from food contact materials into food have also been investigated.^1,2)^ However, these studies are from a human health perspective.

The number of dogs and cats in Japan in 2020 is estimated to be 18 million and the percentage of households that own dogs and cats is approximately 10%.^3)^ Due to increased demands for COVID-19-associated isolation, the number of new pets owned have been the highest in the past five years. Pet owners consider pets to be family members, and according to a survey in 2007 by the Ministry of Agriculture, Forestry and Fisheries and the Ministry of the Environment, the most important factors when purchasing pet food were safety (36.6%) and price range (25.6%) for dogs, and pet preference (30.8%) and safety (26.0%) for cats. Japan has an Act on Ensuring the Safety of Pet Food, which regulates ingredient standards and production methods for pet food, but there are no regulations governing the safety of pet tableware. In this study, additives contained in pet tableware are identified by mass spectrometry and their potential for migration is examined using simulated saliva as the carrier.

## EXPERIMENTAL

### Pet tableware

Plastics, ceramics, pottery, and stainless steel are generally used as materials for pet tableware. A survey conducted in February 2021 at nine retail pet stores, home centers, and discount stores in eastern Tottori Prefecture showed that the number of pet tableware items displayed by material was 341 for plastics, 173 for porcelain/ceramics, and 214 for stainless steel. Among the plastics, 168 were made of melamine formaldehyde resin (MF), 121 of polypropylene (PP), and 15 of polystyrene (PS). In this study, MF and PP pet tableware were the subjects of study, and the samples were purchased in June 2021 and January 2022 in Tottori City.

### Chemicals

Irgafos 168 and Erucamide were purchased from Tokyo Chemical Industry Co. (Tokyo, Japan). Acetone, methanol, chloroform, sodium chloride, potassium chloride, sodium sulfate, ammonium chloride, urea, lactic acid, sodium hydroxide, and 2-propanol were purchased from Kanto Chemical Co. (Tokyo, Japan). Formic acid was purchased from Fluka (Charlotte, NC, USA). Water was generated by PURELAB frex-3 (ELGA, High Wycombe, UK). These reagents were used without further purification. Stock solutions of Irgafos 168 and Erucamide were prepared by dissolving 0.1 g of reagents in 100 mL of acetone and methanol, respectively. Working solutions were prepared by diluting the stock solutions with methanol.

### Chromatography/mass spectrometry

In this study, a gas chromatograph/mass spectrometer (GC/MS) system and two liquid chromatograph/mass spectrometer (LC/MS) systems were used. The GC/MS system consisted of TraceGC Ultra and ion trap MS Polaris Q (ThermoFisher, Waltham, MA, USA). One of the LC/MS systems consisted of ExionLC and a hybrid (quadrupole and time-of-flight) tandem MS X500R (SCIEX, Framingham, MA, USA), which had a mass accuracy of 2 ppm, was used for structural analysis. Another LC/MS system consisted of 1260 Infinity (Agilent, Santa Clara, CA, USA) and a triple quadrupole MS API2000 (SCIEX) was used for quantitation.

### Material tests

In the Japanese regulations, the recommended sample weight for material tests is from 0.5 to 1 g. In the material tests, pet tableware was cut into 2 cm squares using K-100 BANDSAW (Hozan, Osaka, Japan) as the cutting machine. The MF sample and the PP sample were immersed respectively in 1 M hydrochloric acid and chloroform in 100 mL Erlenmeyer flasks and stored at room temperature for 4 weeks. The solvents in which the samples were immersed were filtered through PTFE membrane filters (Advantec, Tokyo, Japan) and diluted 100-, 1000-, and 10,000-fold with acetone and methanol to produce the material test solutions. The acetone diluents were analyzed using GC/MS. The injection volume was 1 μL. The GC column was HP-5MS UI (Agilent, 30 m×0.25 mm, 0.25 μm film thickness). The GC inlet temperature was 250°C, and helium was used as carrier gas at a flow rate of 1 mL/min. The oven temperature programs were as follows: 60°C (2 min hold), 320°C (10°C/min, 10 min hold). Electron ionization at 70 eV was used. The ion source temperature was 250°C. Scan mode acquisitions were carried out in a range of *m*/*z* 20–800.

The methanol diluents were analyzed by an LC/MS system consisting of ExionLC and X500R. Triart C18 (YMC Co., LTD., Kyoto, Japan, 5 cm×2 mm, 5 μm) was used as LC column. Mobile phases were (A) 20 mM formic acid aquatic solution and (B) 20 mM formic acid methanolic solution. The flow rate was 0.2 mL/min. The LC gradient condition was represented as changes in the ratio of A as follows: A 95% (0 min), 95% (3 min), 0% (13 min), 0% (23 min), 95% (23.1 min), 95% (28 min). The injection volume was 5 μL. Electrospray ionization (ESI) was used, and the analyses were carried out in both positive and negative ion modes. The desolvation temperature of the ion source was set at 300°C. The nebulizer gas and heater gas were 50 and 80 psi, respectively. Data dependent acquisition was carried out for the top ten precursor ions with strongest signal intensities from *m*/*z* 100 to 1000.

### Optimization of instrumental conditions for the targeted analysis

Quantitative analyses were carried out by using 1260 Infinity and API2000. Mass spectra were acquired by infusion using a syringe pump Model 11 (Harvard Apparatus, Holliston, MA, USA). Electrospray ionization (ESI) and atmospheric pressure photoionization (APPI) running in the positive-ion mode were examined. A krypton discharge lamp (Cathodeon, Cambridge, UK) was used for APPI. APPI involved the use of toluene as a dopant for ionization at a flow rate of 50 μL/min. The concentrations of working solutions were 1 μg/mL for Irgafos 168 and 0.1 μg/mL for Erucamide. The scan range for Q1 was *m*/*z* 400–700 for Irgafos 168 and *m*/*z* 100–400 for Erucamide. The precursor ions were examined, and selected reaction monitoring (SRM) conditions were optimized. Ion source temperature, nebulizer gas pressure, and heater gas pressure were respectively set at 450°C, 40 psi, and 80 psi. Ion spray voltage for ESI and ion transfer voltage for APPI were 5500 and 1170 V, respectively.

LC column was TSK gel ODS-100 V (TOSOH corp., Tokyo, Japan, 15 cm×2 mm, 3 μm). Injection volume was 5 μL. Column oven temperature was 40°C. Three mobile phase gradient conditions were examined. Condition I was as follows: flow rate, 0.2 mL/min; mobile phase A, water; mobile phase B, methanol; gradient in changes of A, 80% (0 min), 80% (1 min), 0% (6 min), 0% (25 min), 80% (25.1 min), 80% (30 min). Condition II was as follows: flow rate, 0.2 mL/min; mobile phase A, 0.1% (v/v) aqueous formic acid solution; mobile phase B, methanol. The gradient applied was the same as that for Condition I. Condition III was as follows: flow rate, 0.15 mL/min; mobile phase A, 0.1% (v/v) formic acid aquatic solution; mobile phase B, methanol; gradient in change of A, 80% (0 min), 80% (1 min), 0% (30 min), 80% (30. 1 min), 80% (35 min).

### Migration tests by shaking

Mouthing and licking are common ways for pets to eat. In spite of this, there are limited studies of experimental designs for evaluating the migration of chemical substances during periods when pets are eating. Wooten and Smith evaluated the migration of phthalates by immersing pet toys in simulated saliva and shaking them.^4)^ In present study, the migration tests were designed to take into account the area where pets lick the tableware and their mealtimes. PP tableware was cut into 4 cm squares using K-100 BANDSAW. The sample was placed in a glass 50 mL sample tube and 10 mL of simulated saliva was added. The simulated saliva was prepared according to the method by Fiala^5)^ and consisted of 2.25 g of sodium chloride, 0.15 g of potassium chloride, 0.15 g of ammonium chloride, 0.1 g of urea, 1.5 g of butyric acid, and 500 mL of water. The simulated saliva was adjusted to pH 6 and 8 by adding an aqueous sodium hydroxide solution. Sample tubes with caps were shaken with a shaker (Cute Mixer 100, EYELA, Tokyo, Japan) at 1000 rpm for 10 min. Analytes were extracted from the simulated saliva by solid phase extraction (SPE).

### Solid phase extraction

Because the samples for the migration tests contained a significant amount of salt, it cannot be introduced directly into the LC/MS system. To remove the salt, purification by SPE was considered. Because both Irgafos 168 and Erucamide are low polar compounds, Sep-Pak C18 Plus Short (Waters, Milford, MA, USA) was used for a SPE cartridge. Water was used to wash the salt from SPE cartridges and methanol was used as the eluent. The elution profile of the analytes from SPE cartridges was initially examined to determine the amount of eluent required for elution. Ten milliliters of water spiked with 0.5 mL of a 10 μg/mL working standard solution was passed through SPE cartridges that had been preconditioned by the sequential treatment with 5 mL of methanol and 5 mL of water. Ten milliliters of the eluate were fractionated into 1 mL portions, and each portion was analyzed by LC/MS. To determine the solution composition that can be used to rinse hydrophobic analytes adsorbed to the glassware, various compositions of 5 mL aqueous methanol (10, 20, 30, 40, 50, and 60%) were used to rinse the glassware and also loaded on the SPE cartridges adsorbing the analytes, and each eluate was analyzed by LC/MS.

### Method validation

The standard deviation for seven repetitive analyses of working solutions at a concentration of 0.03 μg/mL that gave a signal-to-noise ratio of approximately 10 was determined. This standard deviation was used to determine the detection limit. To a 10 mL portion of simulated saliva prepared at pH 8, 0.5 mL of 10 μg/mL working solution was added and loaded on SPE cartridges. The sample containers were rinsed twice with 5 mL of rinse solution, and the rinse solutions were also loaded on the cartridges. After washing the cartridges with 5 mL of water, the analytes were eluted with 9 mL of methanol. The eluates were combined with 1 mL water and then introduced to the LC/MS analyses.

### Sample analysis

**Figure 1:**
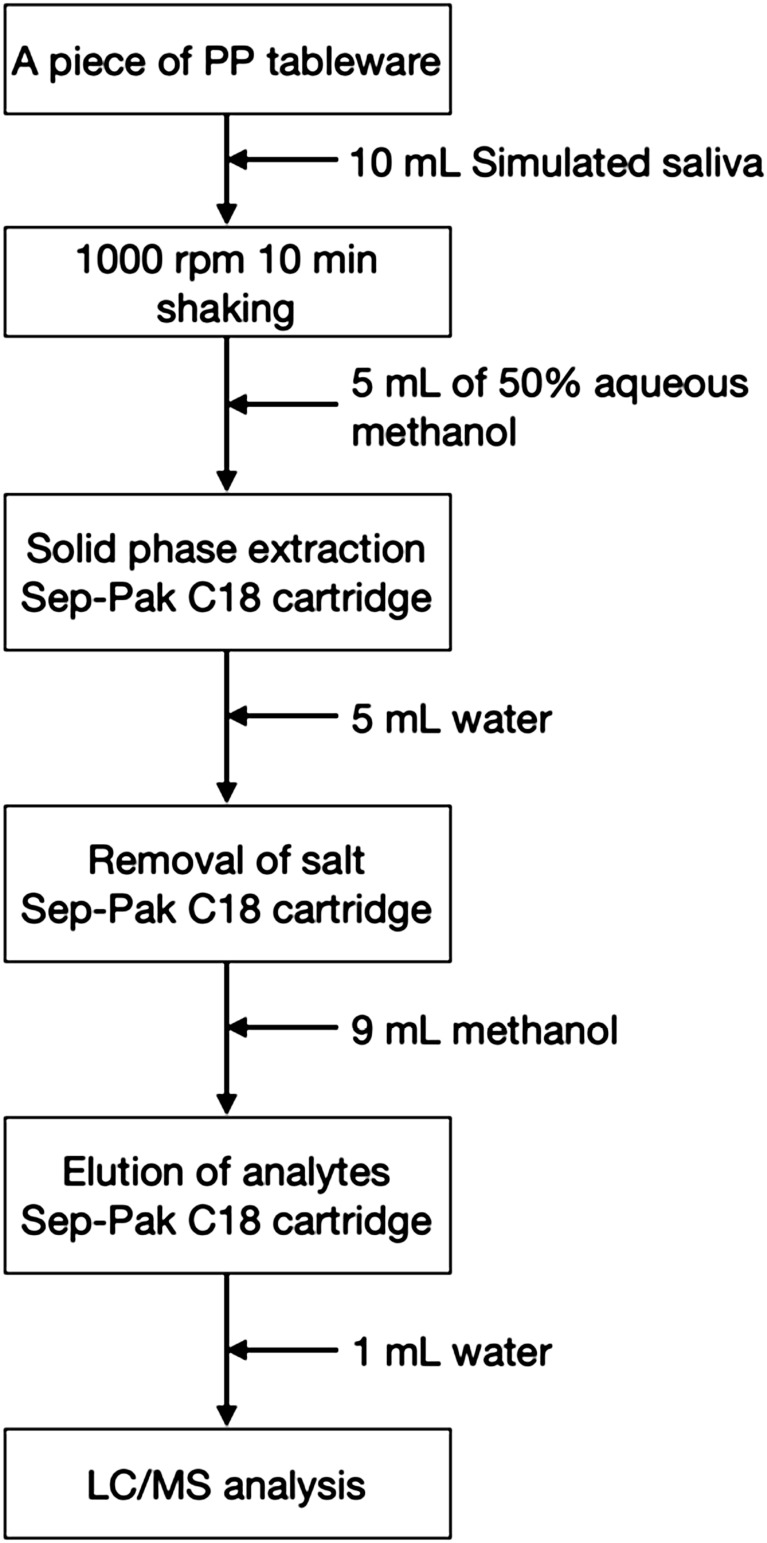
Fig. 1. Scheme for the established pretreatment procedure.

Migration tests were conducted on five types of PP pet tableware purchased from four pet stores or home centers in January 2022. All samples were cut into 4 cm^2^ squares. Elution tests were also performed on a blank sample with 10 mL of simulated saliva adjusted to pH 8. The final preparation procedure is shown in Fig. 1. Each sample was repeatedly extracted by shaking three times. A sample with a shaking extraction time of 30 min and a sample with simulated saliva at pH 6 were also examined.

## RESULTS AND DISCUSSION

### Material tests

**Figure 2:**
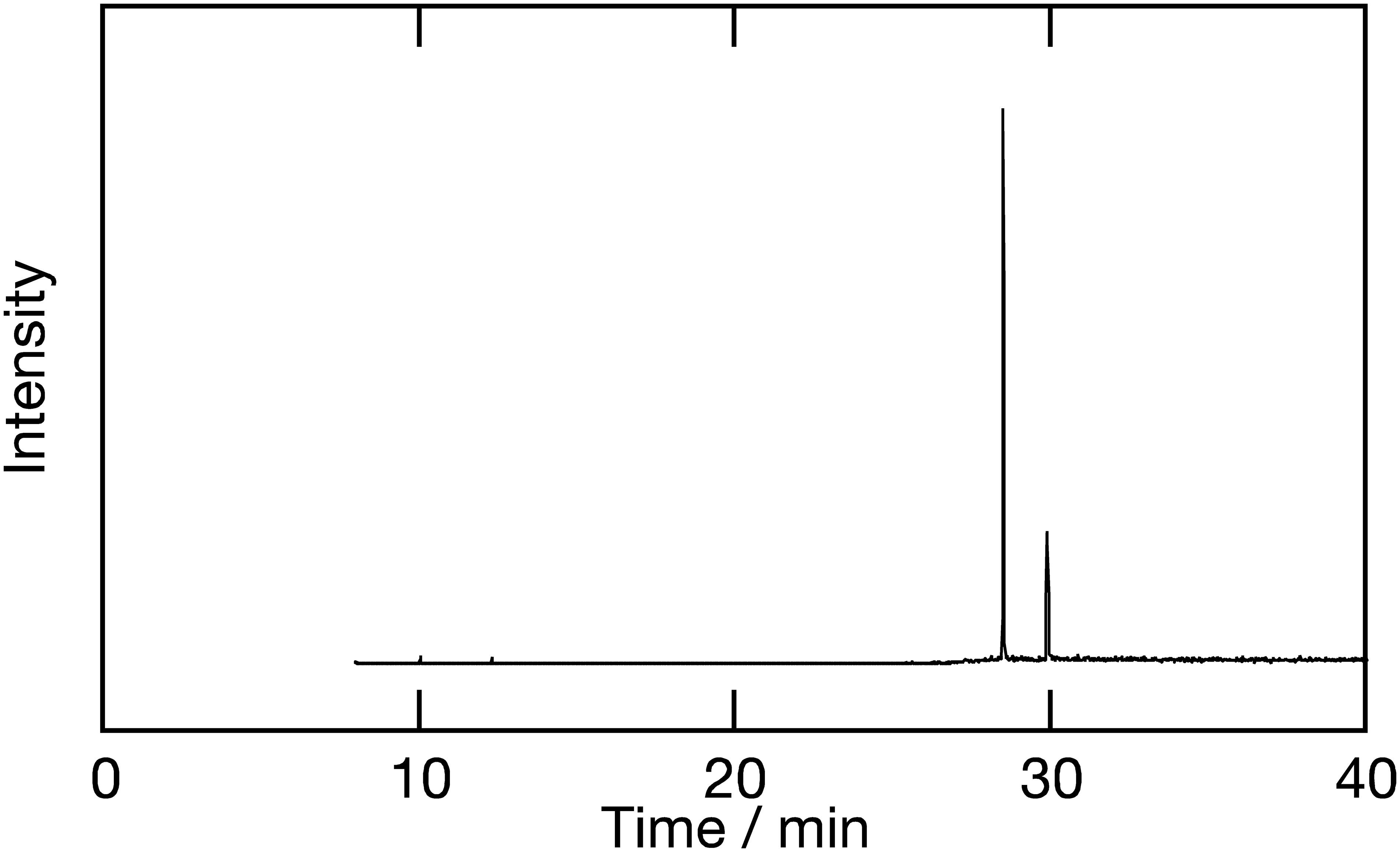
Fig. 2. Extracted ion chromatogram in the range of *m*/*z* 600 to 700 by the GC/MS analysis of the PP material test sample.

**Figure 3:**
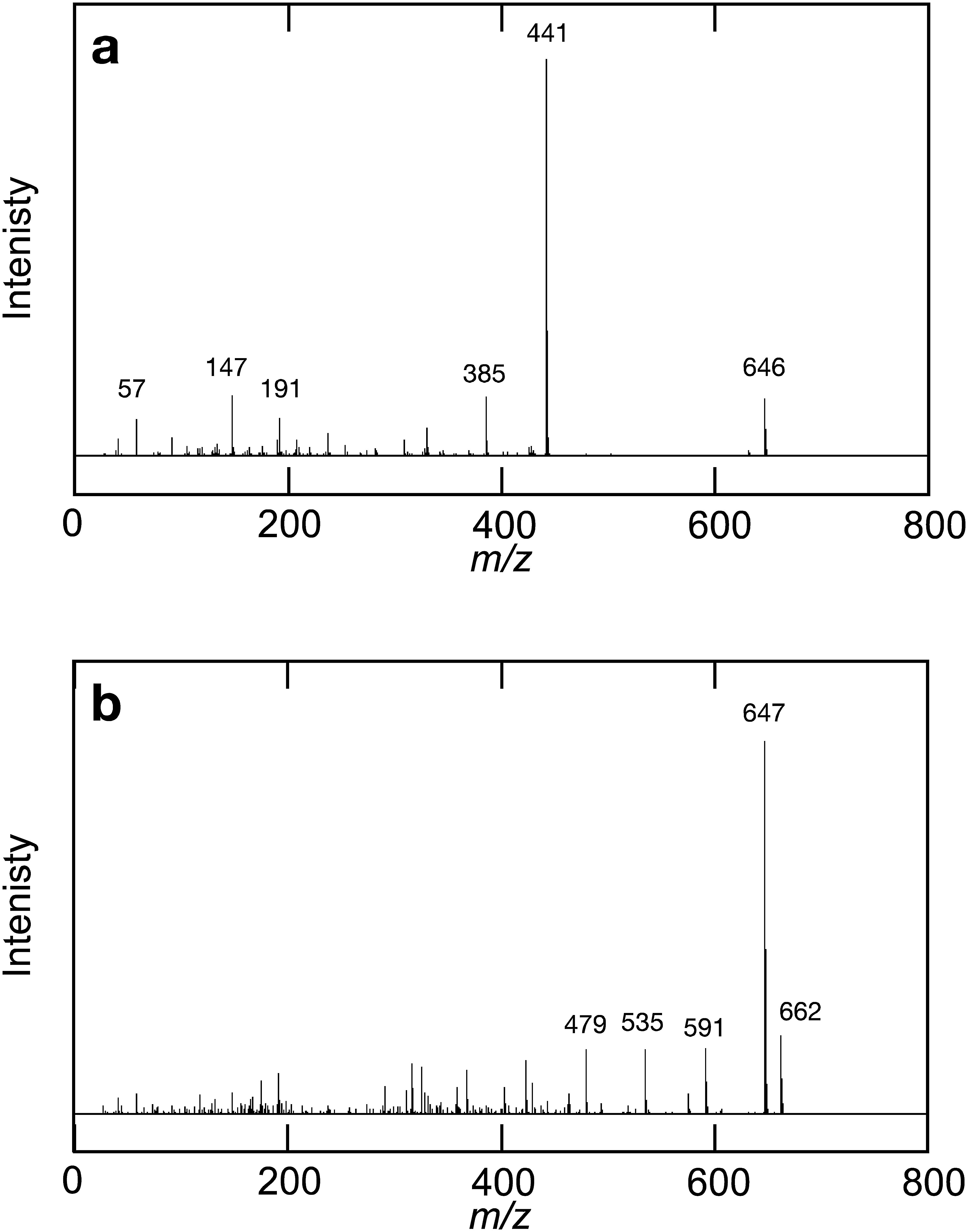
Fig. 3. Mass spectra of the peaks that appeared at 28.5 min (a) and 29.9 min (b) by GC/MS analysis of the PP material test sample.

The data of the material test samples were compared with that of the solvent in order to identify signals corresponding to the material test samples. Figure 2 shows an extracted ion chromatogram from *m*/*z* 600 to 700 acquired from the GC/MS analysis of the PP material test samples. These peaks were detected only from the PP material test samples. The base peak of the peak detected at 28.5 min was *m*/*z* 441, and other ions such as *m*/*z* 57 (12%), 147 (16%), 191 (10%), 385 (14%), and 646 (13%), were detected. The base peak of the peak detected at 29.9 min was *m*/*z* 647, and other ions such as *m*/*z* 57 (5%), 147 (5%), 191 (10%), 316 (12%), 367 (13%), 423 (14%), 479 (20%), 535 (18%), 591 (18%), and 662 (21%), were also detected. Figures 3a and 3b show the mass spectra of the samples. Several product ions with constant *m*/*z* 56 intervals were observed, suggesting that the structures had multiple *tert*-butyl groups. No corresponding mass spectra for them were found in a library search, but similar spectra had been reported by JEOL.^6)^ Because antioxidant Irgafos 168 was a suspected substance, a standard solution of Irgafos 168 was prepared and analyzed in the same manner, and the same peak as the material tests was obtained at 28.5 min. The peak found at 29.9 min was considered to be an oxidized form of Irgafos 168.

**Figure 4:**
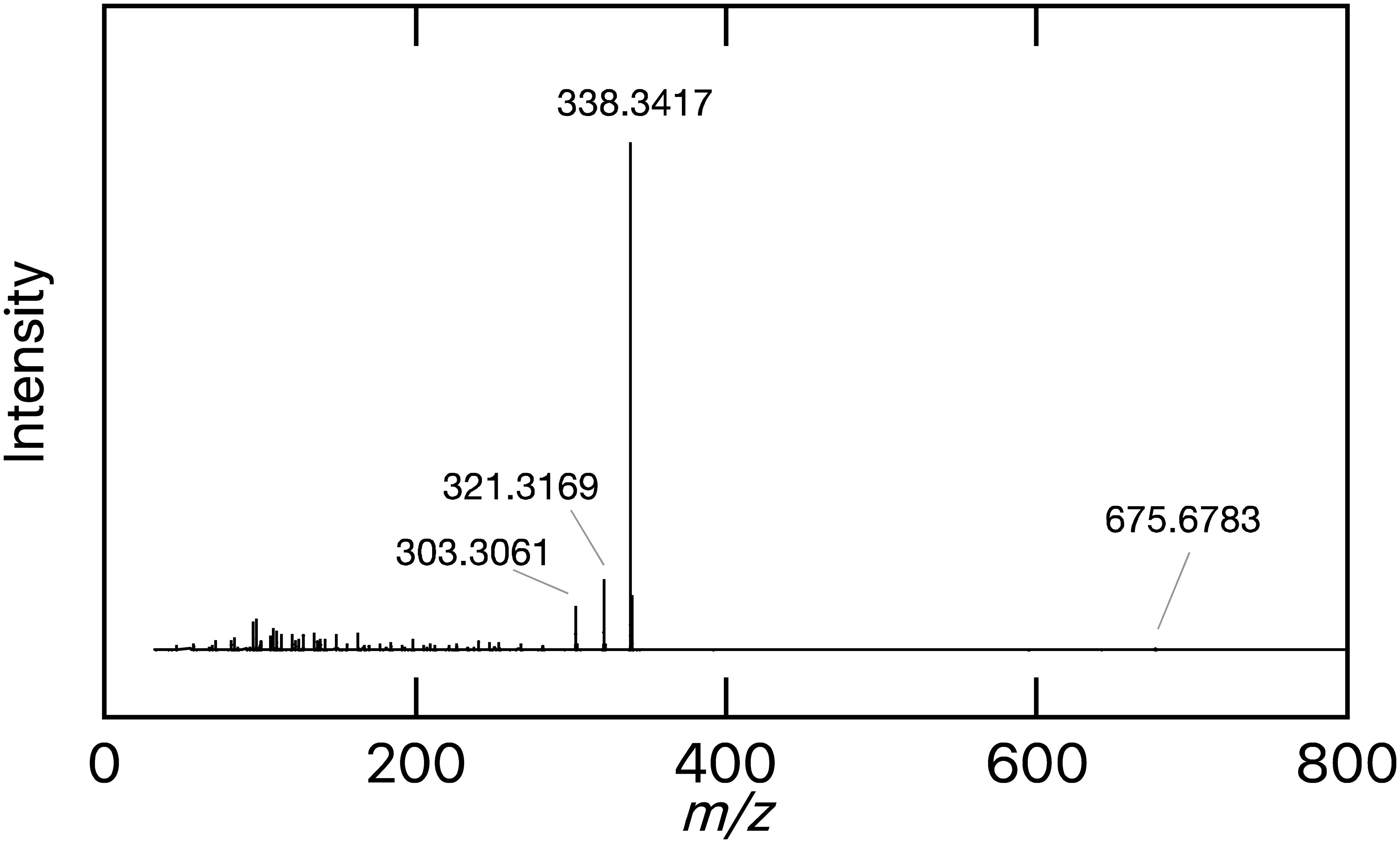
Fig. 4. Product ion spectrum of the peak with the strongest intensity by LC/MS analysis of the PP material test sample.

LC/MS analysis of the material test samples in the positive ion mode provided a peak with *m*/*z* 675.6783 as a specific ion with the strongest intensity. The *m*/*z* values of the product ions generated from the precursor ion of *m*/*z* 675.6783 were *m*/*z* 303.3061, 321.3169, and 338.3417. Figure 4 shows the mass spectrum. The compositional formula of the precursor ion was estimated to be C_44_H_86_N_2_O_2_^+^, because the corresponding compositional formulas of the product ions are C_22_H_39_^+^, C_22_H_41_O^+^, and C_22_H_43_NO^+^, respectively. Because the compositional formula of the precursor ion corresponded to two parts of C_22_H_43_NO^+^, it was reasonable to consider this as a dimer of C_22_H_43_NO^+^. Given that C_22_H_39_^+^ and C_22_H_41_O^+^ were generated from the fragmentation of C_22_H_43_NO^+^, the same fragmentation was found in the MassBank record (accession # MSBNK-Fiocruz-FIO00883), hence indicating the presence of a slip agent, Erucamide. Because MF did not provide a sufficient signal, Irgafos 168 and Erucamide for PP tableware were selected for further study.

### Optimization of instrumental conditions for the targeted analysis

To develop a method for the simultaneous analysis of the two targeted substances, the ionization of each was examined. GC/MS is frequently used for analyses of additives in polyolefin polymers, but fatty acid amides have been reported to show a poor sensitivity.^7)^ The ions detected in the positive ion ESI and APPI by infusion analyses using API2000 and their product ions are listed in Table 1. Irgafos 168 generated protonated molecules in both ionization techniques, however, Erucamide did not generate any protonated molecules in ESI. An in-source fragment [M−H_2_O−NH_3_+H]^+^ and an adduct [M+Na]^+^ were dominant in ESI. In addition, the product ion options were limited. On the other hand, Erucamide produced protonated molecules in APPI, and several product ions were obtained, so a simultaneous analysis in the positive ion mode of APPI was considered. Optimized SRM conditions in the positive ion APPI were determined and the results are shown in Table 2.

**Table 1:** Table 1. Ions produced by infusion analysis from the analytes.

Analyte	Ionization	Ionic form of precursor ion	*m*/*z* value of product ion
Irgafos 168	ESI+	[M+H]^+^	57, 147, 234, 290
	APPI+	[M+H]^+^	57, 147, 234, 290
Erucamide	ESI+	[M−H_2_O−NH_3_+H]^+^	43
		[M+Na]^+^	n/a*
	APPI+	[M+H]^+^	43, 57, 69, 321

*n/a, not available.

**Table 2:** Table 2. Optimized SRM conditions.

	Q1	Q3	DP*/V	CE**/V
Irgafos 168	647	147	71	81
	647	57	71	53
Erucamide	338	321	16	23
	338	43	16	67

*DP, declustering potential; **CE, collision energy.

**Figure 5:**
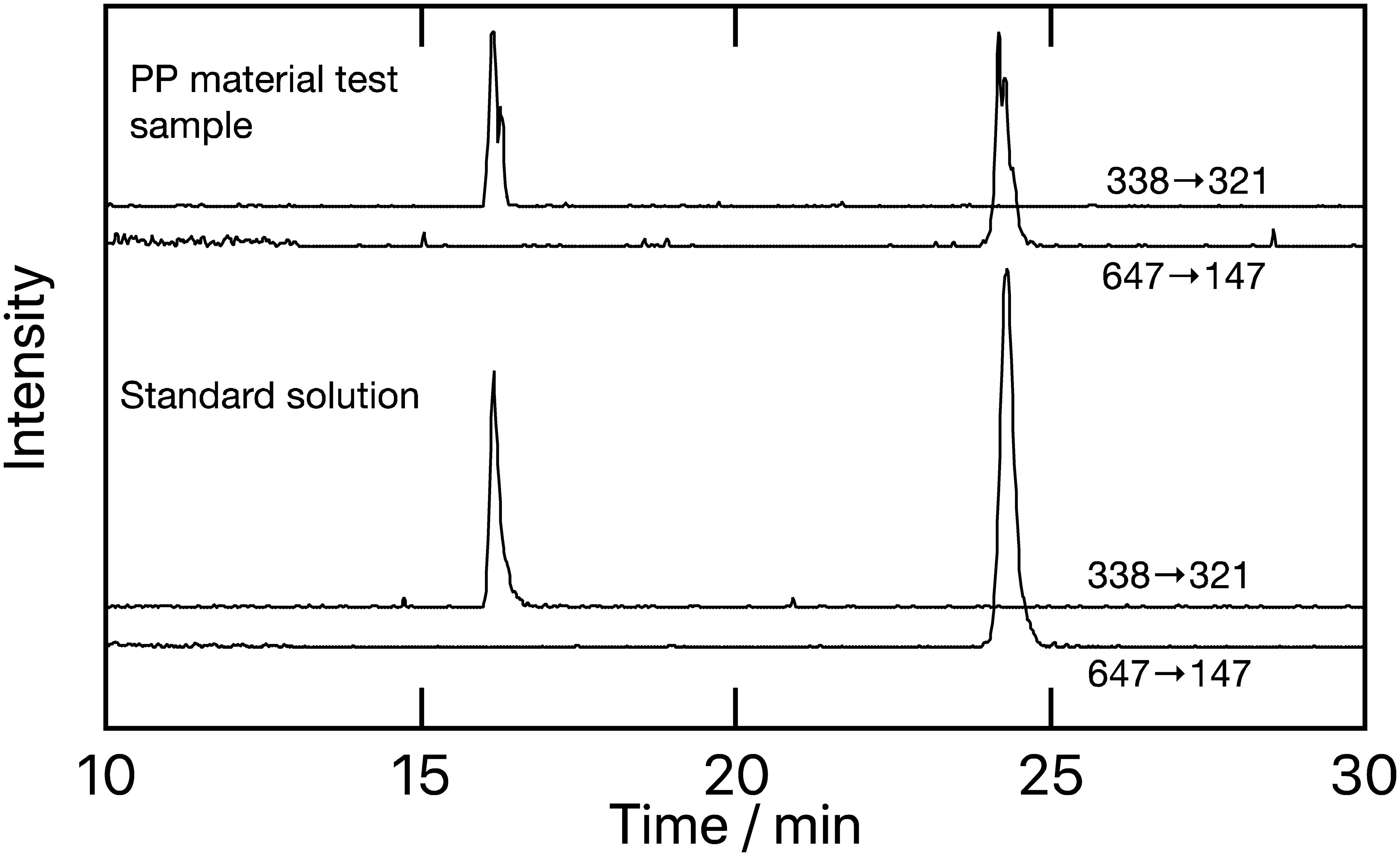
Fig. 5. SRM chromatograms of the PP material test sample and standard solution by LC/MS analysis.

**Figure 6:**
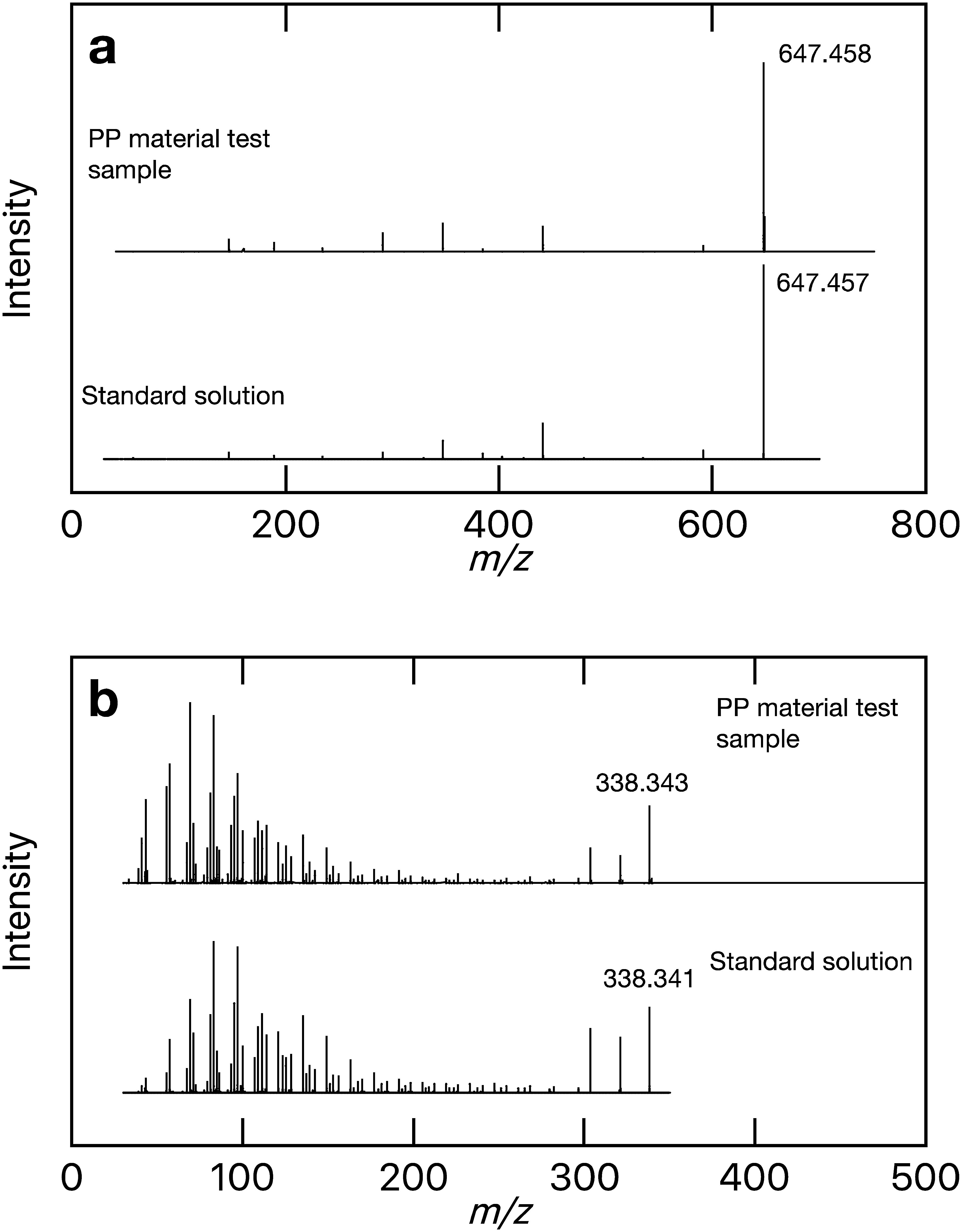
Fig. 6. Product ion spectra of Irgafos 168 (a) and Erucamide (b) included in the PP material test sample and the standard solution by LC/MS analysis.

Chromatographic conditions examined indicated that Irgafos 168 and Erucamide respectively eluted at 24 min and at 16.1 min under gradient conditions I and II. Irgafos 168 and Erucamide suspected to be contained in PP by the material test were confirmed to be present under this LC/MS analysis (Fig. 5). As additional confirmation, the product ion spectra of each peak acquired by X500R are shown in Fig. 6. Because the sensitivity increased under the condition where the mobile phase flow rate was reduced to 0.15 mL/min, the final condition for quantitative analyses was set to condition III.

### Solid phase extraction

**Figure 7:**
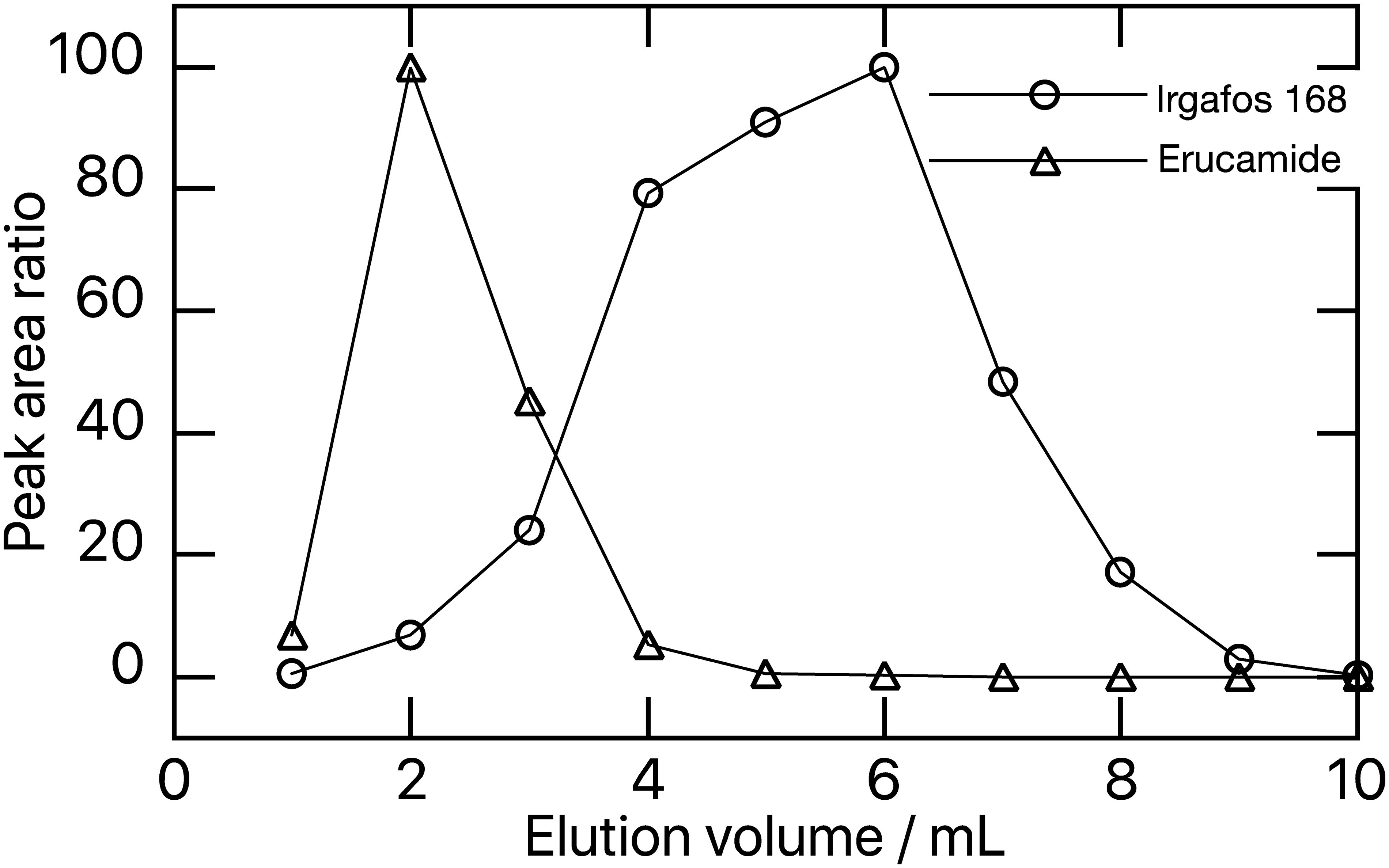
Fig. 7. Elution profiles of analytes from a solid-phase extraction cartridge using methanol as an eluent.

Figure 7 shows the elution profile of analytes loaded on the SPE cartridges when they were eluted with 10 mL of methanol. Octanol–water partition coefficients, log *K*_ow_, estimated by EPI Suite^8)^ were 18.1 for Irgafos 168 and 8.4 for Erucamide, indicating that Irgafos 168 requires more eluent than Erucamide. In the examination of the wash solutions, no elution of the analytes was observed in any of the methanol ratio solutions examined.

### Method validation

The detection limits were determined to be 0.019 μg/mL for Irgafos 168 and 0.022 μg/mL for Erucamide based on the standard deviation of seven analyses of 0.03 μg/mL standard solution. To 10 mL of simulated saliva solutions adjusted to pH 8, 0.5 mL of 10 μg/mL working standard solution were added and loaded on preconditioned solid-phase cartridges. Glassware was rinsed with 60% methanol, and the wash solution was also loaded onto the cartridge. The cartridges were washed with 5 mL of water, and the analytes were eluted with 9 mL methanol. This procedure was performed in triplicate to obtain the recovery rates. The recoveries from this procedure were as low as 54–62% for Irgafos 168 and 22–34% for Erucamide, suggesting that the analytes eluted with 60% methanol rinse solution in the case of simulated saliva conditions. Therefore, the rinse solutions were changed to 50% methanol and rinsing PP and the glassware resulted in recoveries of 82–87% for Irgafos 168 and 100–107% for Erucamide, making it a more reliable analytical method.

### Migration of analytes from pet tableware into simulated saliva

The analytes were below the detection limit in all of the samples with repeated extractions, with longer shaking time, and with simulated saliva at pH 6. The amounts of substance migrated into simulated saliva from pet tableware that were detectable in the current study are greater than 0.19 μg for Irgafos 168 and 0.22 μg for Erucamide. According to European Chemical Agency (ECHA) REACH registered substance factsheets,^9)^ no acute toxicity has been reported in rats at oral doses of 5–6 g/kg bw. Concerning repeated dosing, no observed effect concentrations (NOECs) have been reported to be 147–1000 mg/kg bw (rats). To evaluate the quantities related to safety standards, these NOECs, a safety factor of 100 due to species differences, and body weights of 5 kg for dogs and 4 kg for cats, were used. In addition, the number of meals was set to two times per day. The intakes of the target substances for which no effect was verified were converted into safety standards. The corresponding intake per meal would be 2.94–25 mg, which is more than sufficient for this study. Therefore, it is improbable that analytes that are migrated from PP tableware would have any adverse effects on dogs and cats.

## Notes

Mass Spectrom (Tokyo) 2023; 12(1): A0119
